# Health Status of Traffic Police Personnel in Brahmapur City

**DOI:** 10.4103/0970-0218.45380

**Published:** 2009-01

**Authors:** DM Satapathy, TR Behera, RM Tripathy

**Affiliations:** Department of Community Medicine, MKCG Medical College, Berhampur, Orissa, India

## Introduction

Health is not something that one possesses as a commodity, but connotes rather a way of functioning within one's environment (work, recreation, and living). The work environment constitutes an important part of man's total environment, so health to a large extent is affected by work conditions.(1) Though several types of environment exist, it is the physical environment, which plays an important bearing on health. Air, noise, heat, radiation, etc., are the main sites of environment pollution and this is more so in urban areas.

Occupational environment too plays a major role on the health of the exposed. The health hazards get more severe when the duration of exposure increases. This fact is more important in situations as the personnel engaged in traffic duty. These personnel have to undergo physical strain in an environment polluted by fumes, exhaust of vehicles, use of blowing horns, blow of dust in the air by a speeding vehicle, etc. The personnel also pursue a near-sedentary type of work as they only stand at one place for long hours or just walk a few meters, only when necessity arises. The aforementioned factors pose as a health hazard. Still little has been done to assess their health status and suggest preventive measures for the upliftment of their health. A study was thereby conducted to assess the health status of the traffic police personnel of the Berhampur city and to find out the related risk factors so that appropriate preventive measures can be recommended for safe guarding the health.

## Materials and Methods

The study was a cross sectional study, conducted during 15^th^ May 2004 to 15^th^ July 2004 in Berhampur with prior permission from the superintendent of police of Brahmapur circle. The Brahmapur police division (urban) caters to the municipality area and the adjoining villages with a total population of about 3.6 lakh as on 2001. However, the division has only 48 personnel engaged as constables on traffic duty. All the constables were thereby taken as the study population. Health status was assessed by conducting appropriate anthropometrical, clinical, and laboratory examination of each subject. Peak expiratory flow rate was taken as best of three readings at one sitting with the help of Wright's peak flow meter. Fasting blood sugar was conducted by using Glucose Oxidase method after taking the blood sample in the early hours of morning before joining their duty.(2)

## Result

Out of total 48 traffic police personnel 43 (89.6%) were males and 5 (10.4%) were females. Majority (89.6%) were between 30-50 years. Tobacco chewing was the most common (48%) form of addiction, followed by alcohol in 20.8% of study subjects. It was observed that 38.3% of subjects were overweight and 8.5% were obese (according to their BMI). The study revealed that 25% of study subjects were hypertensive. Among the persons who were obese (BMI >25), 31.8% were hypertensive and among normal/thin (BMI <25), 19.2% were hypertensive and this difference of BMI and hypertension was not significant but there was a positive correlation (*r*=0.4) between BMI and hypertension. Majority (66.6%) of subjects had a PEFR value between 300 and 500 liters/min, 8.3% had below 300 Lt/min and 25% had above 500 Lt/min. The study revealed a negative correlation (*r*=-0.6) between PEFR and BMI.

Among the different morbidity patterns studied in the traffic police personnel, anaemia was observed in 43.75%, musculoskeletal disorders in 27.08%, hypertension in 25%, eosinophillia in 18.75% [Table 1]. In only 16% of subjects' respiratory disorders like rhinorrhoea, chronic bronchitis, pharyngitits, etc., were observed. Only 2 persons had varicose veins of legs, which were detected by Trendlenberg's test. This may be due to prolonged standing hours(2) or may be due to obesity.

**Table 1:** Different morbidities detected

Type of morbidity	Number (%)
Anemia	21 (43.75)
Musculoskeletal disorders	13 (27.08)
Hypertension	12 (25)
Eosinophilia	9 (18.75)
Respiratory disorders	8 (16.66)
Gastrointestinal disorders	7 (14.58)
Diabetes mellitus	3 (6.25)
Dermatoses	3 (6.25)
Allergic conjunctivitis/Visual difficulties	3 (6.25)
Varicose veins	2 (4.16)

## Discussion

This study revealed there were no pre-placement examination of these traffic police personnel regarding pulmonary function, and mental status nor are they being periodically examined for their health status to detect any morbidity. Peter *et al*. in their study of acute health effects of exposure to high levels of air pollution in Eastern Europe have found that inhalable particles are more closely associated with adverse health effects than gaseous pollutants.(3)

Hence, like in industries, the traffic police personnel should be periodically examined for their health status.
